# Attributable burden of steatotic liver disease on cardiovascular outcomes in Asia

**DOI:** 10.1016/j.jhepr.2025.101479

**Published:** 2025-06-06

**Authors:** Szu-Ching Yin, Yi-Ting Chen, Wei-Ting Chang, Tzu-I Chen, Tsai-Hsuan Yang, Xia-Rong Liu, Chia-Wei Huang, Yu-Wei Chen, Mei-Hsuan Lee

**Affiliations:** 1 Institute of Clinical Medicine, National Yang Ming Chiao Tung University, Taipei, Taiwan; 2 Division of Cardiology, Department of Internal Medicine, Chi Mei Medical Center, Tainan, Taiwan; 3 School of Medicine and Doctoral Program of Clinical and Experimental Medicine, College of Medicine and Center of Excellence for Metabolic Associated Fatty Liver Disease, National Sun Yat-sen University, Kaohsiung, Taiwan; 4 Advanced Therapeutics Research Center, National Yang Ming Chiao Tung University, Taipei, Taiwan; 5 Department of Pulmonary Hypertension, Cardiovascular Center, Taichung Veterans General Hospital, Taichung, Taiwan; 6 Department of Post-Baccalaureate Medicine, College of Medicine, National Chung Hsing University, Taichung, Taiwan; 7 Cardiovascular Research Center, College of Medicine, National Chung Hsing University, Taichung, Taiwan; 8 Master of Public Health Program, National Yang Ming Chiao Tung University, Taipei, Taiwan

**Keywords:** long-term risk, fatty liver disease, cardiometabolic risk factor, major adverse cardiovascular events, noncommunicable diseases

## Abstract

**Background & Aims:**

The associations between metabolic dysfunction-associated steatotic liver disease (MASLD) and specific cardiovascular events, as well as their attributable burdens, remain inconsistent and underexplored within a single population. This large-scale prospective cohort evaluated the associations between MASLD and various cardiovascular outcomes. Two additional steatotic liver disease (SLD) subtypes – MASLD with increased alcohol consumption (MetALD) and alcohol-related liver disease (ALD) – were also evaluated.

**Methods:**

We included 303,589 adults aged ≥30 years from Taiwan who underwent health examinations between 1997 and 2013. MASLD was defined by ultrasound-detected steatosis, limited alcohol intake, and ≥1 cardiometabolic risk factor. MetALD and ALD were defined based on alcohol intake thresholds and cardiometabolic profiles. Participants were followed until 2020, with outcomes and mortality ascertained via linkage to national registries. Cox proportional hazards models were used to estimate adjusted relative risks (RRs), and population attributable fractions (PAFs) were calculated.

**Results:**

Of the total population, 91,877 (30.3%) had MASLD, 7,490 (2.5%) had MetALD, 5,576 (1.8%) had ALD, and 198,646 (65.4%) did not have SLD. Over a median follow-up of 10.4 years, 162,959 cardiovascular events occurred. The adjusted RR of any cardiovascular diseases was 1.29 (95% CI 1.38-1.31) for MASLD, 1.38 (95% CI 1.34-1.42) for MetALD, and 1.48 (95% CI 1.43-1.53) for ALD. Among all SLD subtypes, MASLD showed the highest RR for myocardial infarction (RR 1.46, 95% CI 1.36–1.56). Findings remained consistent after accounting for liver-related deaths. The PAF for MASLD was 8.07% (95% CI 7.81–8.58). Despite higher risks, MetALD and ALD had lower PAFs due to lower prevalence.

**Conclusions:**

All major SLD subtypes – MASLD, MetALD, and ALD – were associated with increased long-term cardiovascular risk, underscoring the need for early detection and cardiometabolic risk management across the SLD spectrum.

**Impact and implications:**

This large-scale study of 303,589 individuals demonstrates that metabolic dysfunction-associated steatotic liver disease (MASLD) increases the risk of cardiovascular diseases by at least 29%. Cardiovascular risk further escalates across SLD subtypes with higher levels of alcohol consumption. Notably, MASLD was associated with the highest risk of myocardial infarction among all SLD subtypes. By quantifying population burden, we found that 8.07% of cardiovascular events may be preventable through effective MASLD prevention strategies, highlighting the critical role of cardiometabolic risk management. These findings emphasize the need to integrate MASLD identification and prevention into broader cardiometabolic care and public health frameworks.

**Clinical trial number:**

not applicable.

## Highlights

• Patients with MASLD had a 30% higher relative risk for cardiovascular disease than those without SLD.
• The relative risk for cardiovascular disease was increased further for MetALD and ALD.
• MASLD had the greatest population impact, with a PAF of 8.07% for cardiovascular events.
• MetALD and ALD had lower attributable fractions despite higher risks due to lower population prevalence.

## Graphical abstract

**Figure:**
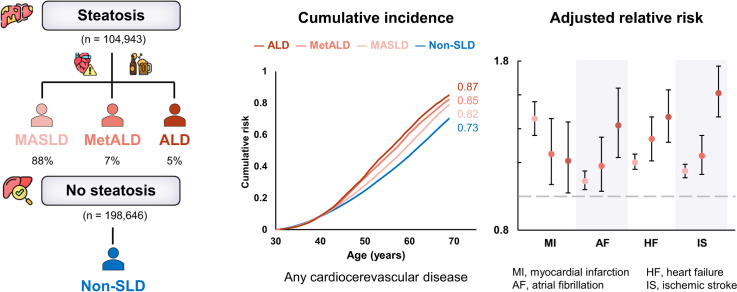

## Introduction

The global obesity crisis, driven by urbanization, sedentary lifestyles, and high-fat diets, has reached alarming levels. Over 1.9 billion individuals are now classified as overweight, with 650 million considered obese.^1^ This surge has led to a dramatic rise in obesity-related diseases, with deaths linked to obesity more than doubling in recent decades.^2^ Alongside this epidemic, the prevalence of steatotic liver disease (SLD), particularly metabolic dysfunction-associated steatotic liver disease (MASLD), has increased dramatically, affecting an estimated 25% to nearly 40% of the global population.^3^^,^^4^ MASLD is now a leading cause of end-stage liver diseases, with cases expected to increase over 21%, from 83.1 million in 2015 to 100.9 million by 2030.^5^

In addition to liver-related complications, cardiovascular disease remains the leading cause of death among patients with MASLD, significantly contributing to their overall health burden.^6^ Evidence suggests MASLD is associated with subclinical atherosclerosis, as indicated by increased coronary calcification scores and calcified coronary plaques.^7^ Given the combination of steatosis and cardiometabolic risk factors, individuals with MASLD are at elevated risk for cardiovascular diseases.^8^ Mendelian randomization studies have further established a causal link between MASLD, atherosclerosis,^9^ and cardiovascular disease,^10^^,^^11^ highlighting the need for targeted management strategies. While meta-analyses have shown associations between MASLD and both fatal and non-fatal cardiovascular diseases,^12^^,^^13^ observational studies have yielded inconsistent results, particularly regarding specific cardiovascular subtypes.[14], [15], [16], [17], [18] Understanding the risks MASLD poses for various cardiovascular events is essential for guiding treatment decisions, yet few studies have thoroughly examined these associations within a single population.

As clinical guidelines increasingly emphasize multidisciplinary care for patients with MASLD,^19^^,^^20^ a comprehensive assessment of its links to specific cardiovascular outcomes is critical for improving management strategies and raising awareness. Moreover, quantifying the proportions of these events attributable to MASLD is essential for informing policy and resource allocation. In this long-term prospective study, we aimed to evaluate the impacts of MASLD and other subtypes of SLD, including MASLD with increased alcohol consumption (MetALD) and alcohol-related liver disease (ALD), on several pre-specified cardiovascular events, estimating the long-term risks and quantifying the proportion of events that could be prevented through SLD prevention strategies.

## Patients and methods

### Study population and data collection

The study cohort consisted of 421,941 adults aged over 30 years who participated in a health screening program operated by a private healthcare institution in Taiwan from 1997 to 2013. After excluding the adults who had prevalent cardiovascular diseases (n = 86,247), we included 307,616 study participants in the subsequent analyses. Detailed participant information and data collection methods have been previously described.^21^ Briefly, all participants underwent comprehensive health assessments, including blood and urine tests, anthropometric measurements, and physical examinations. A standardized questionnaire was used to collect data on lifestyle habits and personal and family medical histories.

### Ethical approval

The study was conducted in accordance with the principle of the Declaration of Helsinki. All participants provided informed consent for the use of their data in biomedical research. The study protocol received approval from the Institutional Review Board of National Yang Ming Chiao Tung University, Taipei, Taiwan.

### Definition of MASLD, MetALD, ALD and reference groups

Hepatic steatosis was diagnosed using high-resolution real-time abdominal ultrasonography performed by board-certified gastroenterologists. Participants lacking adequate ultrasonography data or alcohol consumption records were excluded. Alcohol intake was assessed via self-reported questionnaires at study entry, which included frequency of consumption per week, duration of use (in years), and number of drinks per session (one drink = 150 cc). Participants with steatosis were classified into three subtypes based on their alcohol consumption and cardiometabolic risk profiles: MASLD, MetALD, and ALD. According to the Asian-Pacific guidelines,^22^ limited alcohol intake was defined as less than 20 g/day for men and less than 10 g/day for women; moderate intake as 10–40 g/day for women and 20–50 g/day for men; and excessive intake as >40 g/day for women and >50 g/day for men. Individuals with steatosis and limited alcohol consumption who had at least one cardiometabolic risk factor were classified as having MASLD.^23^ Those with moderate alcohol intake and at least one cardiometabolic risk factor were defined as having MetALD. ALD was defined as steatosis with moderate alcohol intake but no cardiometabolic risk factors, or steatosis with excessive alcohol intake regardless of cardiometabolic status. Participants without ultrasonography-detected steatosis were classified as not having steatotic liver disease (non-SLD) and served as the reference group. In total, 91,877 participants were classified as having MASLD, 7,490 as having MetALD, 5,576 as having ALD, and 198,646 as non-SLD. The study flow is shown in Fig. S1.

### Follow-up and ascertainment of cardiovascular events

We performed computerized data linkage with Taiwan’s National Health Insurance database and National Death Certification system to ascertain cardiovascular events and their vital status. These nationwide registries cover nearly 100% of the Taiwanese population and provide complete, updated, and accurate administrative claim data. Follow-up began on January 1, 1997, and ended on December 31, 2020. We utilized the National Health Insurance Database, identifying patients meeting at least one hospital admission code or with two or more outpatient visits.^24^ The date of the first hospital admission or outpatient visit was used as the incident event date. Cardiovascular events identified using claims data have been reported to be accurate.^25^^,^^26^ Cardiovascular events were identified using ICD-9 and ICD-10 codes, covering cardiovascular disease, cerebrovascular disease, and major cardiovascular events (*e.g.* myocardial infarction, atrial fibrillation, heart failure, and ischemic stroke). Detailed ICD codes are listed in Table S1. The primary outcome was the first occurrence of any cardiovascular events during follow-up.

### Statistical methods

Baseline characteristics were described using absolute numbers and percentages. Categorical variables were compared using chi-squared tests. Participants were followed from enrollment until the occurrence of cardiovascular events, deaths, or the last available follow-up date (December 31, 2020). Incidence rates were calculated by dividing the number of events by person-years of follow-up. Cumulative lifetime risks (ages 30-70 years) for any cardiovascular events were estimated across SLD subtypes. Excessive burdens attributable to MASLD, MetALD, and ALD were assessed by calculating the differences in cumulative incidence between each subtype and the non-SLD reference group at 5, 10, 15, and 20 years of follow-up. Cox’s proportional hazards models were used to derive relative risks (RRs) with 95% CIs, adjusting for potential confounders. The proportional hazards assumption was evaluated by including interaction terms between each subtype and log(time), and no violations were detected. The population attributable fractions (PAFs)^27^ of MASLD, MetALD, and ALD were calculated using the estimated RR and the prevalence of each subtype. To account for competing risks from liver-related deaths (including cirrhosis and hepatocellular carcinoma), we conducted additional analyses using Fine and Gray models and estimated subdistribution hazard ratios (SHRs) with 95% CIs. These analyses provided competing risk-adjusted estimates of the associations between each SLD subtype and cardiovascular outcomes. All statistical significance tests were two-sided, and *p* values <0.05 were considered statistically significant. All analyses were performed using SAS version 9.4 (SAS Institute, Cary, NC).

## Results

### Baseline characteristics and study population

Among the 303,589 participants, 198,646 participants (65.4%) were classified as non-SLD, 91,877 (30.3%) had MASLD, 7,490 (2.5%) had MetALD, and 5,576 (1.8%) had ALD. Compared to the non-SLD group (mean age: 41.0 ± 10.9 years), individuals with SLD (MASLD, MetALD, and ALD) were older, with mean ages of 44.2 ± 11.5, 44.3 ± 10.8, and 44.6 ± 10.2 years, respectively. The proportion of males increased with alcohol intake: 63% in MASLD, 89% in MetALD, and 91% in ALD. Metabolic syndrome was more prevalent in SLD subtypes than in the non-SLD group, affecting 29% of individuals with MASLD, 35% with MetALD, and 37% with ALD. These subtypes also had a higher prevalence of abnormal triglyceride levels and elevated FIB-4 (Fibrosis-4 index), indicating greater liver-related and cardiometabolic risks (Table 1).

**Table 1:** Baseline characteristics of the study population.

Baseline characteristics	Total (n = 303,589)		Non-SLD (n = 198,646; 65.4%)		MASLD (n = 91,877; 30.3%)		MetALD (n = 7,490; 2.5%)		ALD (n = 5,576; 1.8%)	
	n	%	n	%	n	%	n	%	n	%
Age (years)										
Mean ± SD	42.1 ± 11.2		41.0 ± 10.9		44.2 ± 11.5		44.3 ± 10.8		44.6 ± 10.2	
30-<40	160,554	52.9	115,017	57.9	40,390	44.0	3,040	40.6	2,107	37.8
40-<50	69,195	22.8	42,425	21.4	22,708	24.7	2,261	30.2	1,801	32.3
50-<60	44,170	14.5	24,272	12.2	17,399	18.9	1,366	18.2	1,133	20.3
60-<70	23,162	7.6	12,843	6.5	9,182	10.0	667	8.9	470	8.4
≥70	6,508	2.1	4,089	2.1	2,198	2.4	156	2.1	65	1.2
Sex										
Female	153,151	50.4	117,814	59.3	33,955	37.0	855	11.4	527	9.5
Male	150,438	49.6	80,832	40.7	57,922	63.0	6,635	88.6	5,049	90.5
BMI (kg/m^2^)										
Mean ± SD	23.2 ± 3.5		21.7 ± 2.7		26.0 ± 3.1		26.1 ± 3.0		25.8 ± 3.3	
<18.5	20,849	6.9	20,648	10.4	141	0.2	11	0.2	49	0.9
18.5-<23	133,262	43.9	118,634	59.7	12,700	13.8	899	12.0	1,029	18.5
23-<25	66,055	21.8	37,535	18.9	25,297	27.5	1,919	25.6	1,304	23.4
≥25	83,364	27.5	21,789	11.0	53,722	58.5	4,659	62.2	3,194	57.3
Missing	59									
Metabolic syndrome^a^										
No	262,891	86.6	189,701	95.5	64,847	70.6	4,843	64.7	3,500	62.8
Yes	40,698	13.4	8,945	4.5	27,030	29.4	2,647	35.3	2,076	37.2
Smoking										
Never	198,973	66.2	137,701	70.0	58,491	64.3	1,833	24.9	948	17.2
Ever	101,502	33.8	58,886	30.0	32,501	35.7	5,543	75.1	4,572	82.8
Missing	3,114									
Alcohol consumption										
Never	241,931	79.7	162,905	82.0	79,026	86.0	0		0	
Ever	61,658	20.3	35,741	18.0	12,851	14.0	7,490	100.0	5,576	100.0
LDL-C										
Mean ± SD	120.5 ± 33.1		112.4 ± 37.0		121.6 ± 44.0		128.0 ± 34.0		125.1 ± 36.1	
<130	185,380	64.1	133,512	69.7	45,474	53.0	3,635	53.5	2,759	55.9
≥130	103,831	35.9	58,127	30.3	40,371	47.0	3,154	46.5	2,179	44.1
Missing	14,378									
Triglyceride (mg/dl)										
Mean ± SD	121.0 ± 106.1		93.5 ± 61.3		166.3 ± 130.1		210.2 ± 189.0		235.7 ± 259.3	
<150	235,075	77.5	177,636	89.5	51,815	56.4	3,268	43.6	2,356	42.3
≥150	68,379	22.5	20,911	10.5	40,028	43.6	4,220	56.4	3,220	57.7
Missing	135									
FIB-4										
Mean ± SD	0.9 ± 0.6		0.9 ± 0.6		0.9 ± 0.6		1.0 ± 0.8		1.1 ± 1.0	
<1.45	272,431	89.9	178,177	89.7	83,071	90.6	6,543	88.1	4,640	84.1
1.45-<3.25	28,715	9.5	18,956	9.5	8,178	8.9	823	11.1	758	13.7
≥3.25	2,003	0.7	1,425	0.7	392	0.4	65	0.9	121	2.2
Missing	440									
Average use of alcohol (g alcohol/week)										
Mean ± SD	42.5 ± 150.0		34.3 ± 132.3		7.6 ± 23.2		215.4 ± 62.2		674.7 ± 343.5	

ALD, alcohol-associated liver disease; FIB-4, Fibrosis-4 index; LDL-C, low-density lipoprotein-cholesterol; MASLD, metabolic dysfunction-associated steatotic liver disease; MetALD, MASLD and increased alcohol consumption; non-SLD, without steatotic liver disease.

a Metabolic syndrome: three or more of the following criteria: (1) high blood pressure (systolic or diastolic blood pressure ≥130/85 mmHg or use of drugs for hypertension), (2) hyperglycemia (fasting glucose ≥110 mg/dl), (3) hypertriglyceridemia (triglyceride level ≥150 mg/dl), (4) low high-density lipoprotein-cholesterol (men, <40 mg/dl; women, <50 mg/dl), or (5) central obesity (waist circumference ≥90 cm for men, ≥80 cm for women).

### Incidence rates and lifetime risks of cardiovascular diseases for MASLD, MetALD, and ALD

Over a median follow-up of 10.4 years, a total of 162,959 any cardiovascular events occurred, with incidence rates of 6,909.8 and 4,140.1 per 100,000 person-years in the MASLD and non-SLD groups, respectively (Table S2). Participants with MASLD exhibited higher incidence rates for cardiovascular, cerebrovascular, and major cardiovascular subtypes compared to the non-SLD group. Heart failure and ischemic stroke accounted for the largest proportion of events (35.0% and 36.7%, respectively).

**Fig. 1:**
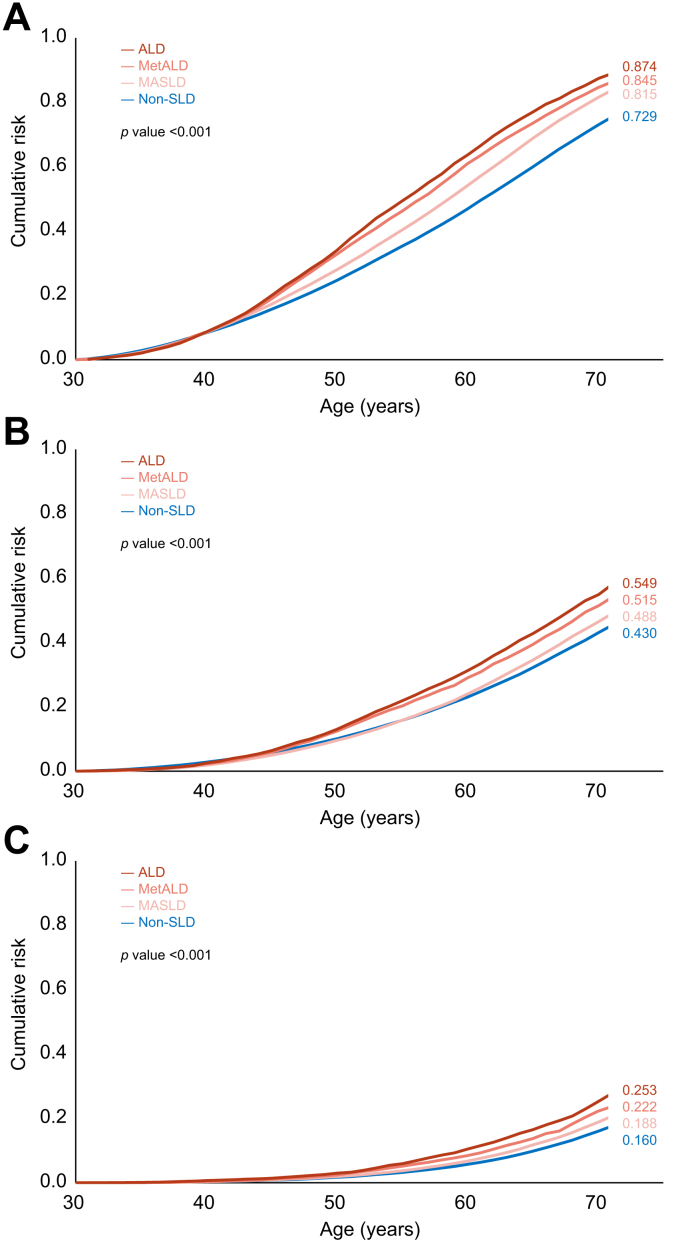
Cumulative lifetime risk of overall cardiovascular diseases. (A) Any cardiovascular disease. (B) Cardiovascular disease. (C) Cerebrovascular disease. Levels of significance: *p <*0.001 (Log-rank test). ALD, alcohol-associated liver disease; MASLD, metabolic dysfunction-associated steatotic liver disease; MetALD, MASLD and increased alcohol consumption; Non-SLD, without steatotic liver disease.

**Fig. 2:**
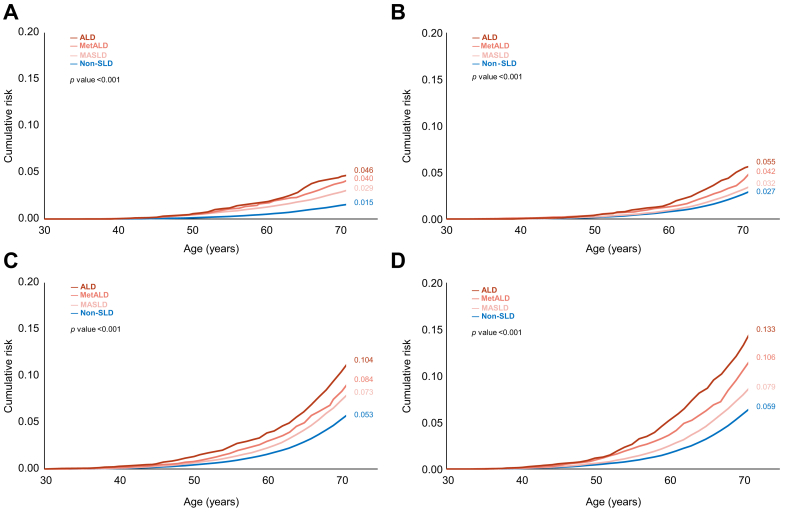
Cumulative lifetime risk of major cardiovascular disease subtypes. (A) Myocardial infarction. (B) Atrial fibrillation. (C) Heart failure. (D) Ischemic stroke. Levels of significance: *p <*0.001 (Log-rank test). ALD, alcohol-associated liver disease; MASLD, metabolic dysfunction-associated steatotic liver disease; MetALD, MASLD and increased alcohol consumption; Non-SLD, without steatotic liver disease.

Cumulative lifetime risk (ages 30-70 years old) estimates revealed significantly higher risks in MASLD compared to non-SLD participants: 49% *vs.* 43% for cardiovascular disease (Fig. 1B, *p <*0.001) and 19% *vs.* 16% for cerebrovascular disease (Fig. 1C, *p <*0.001). Similar trends were observed for myocardial infarction, atrial fibrillation, heart failure, and ischemic stroke (Fig. 2; all *p <*0.001).

Participants with MetALD had even higher incidence rates, reaching 7,898.1 per 100,000 person-years. Their cumulative lifetime risk peaked between ages 50 and 60, with risks of 52% for cardiovascular and 22% for cerebrovascular diseases. Ischemic stroke showed the greatest increase in cumulative incidence, rising by 3% relative to MASLD. Participants with ALD had the highest burden across all groups, with an incidence rate of 8,743.7 per 100,000 person-years. Incidence rates of all cardiovascular events were over twice those observed in the non-SLD group, with myocardial infarction showing the most pronounced difference (3.1-fold higher). Lifetime risks in the ALD group reached 55% for cardiovascular and 25% for cerebrovascular diseases.

### Excessive burden of MASLD, MetALD, and ALD on cardiovascular diseases

**Fig. 3:**
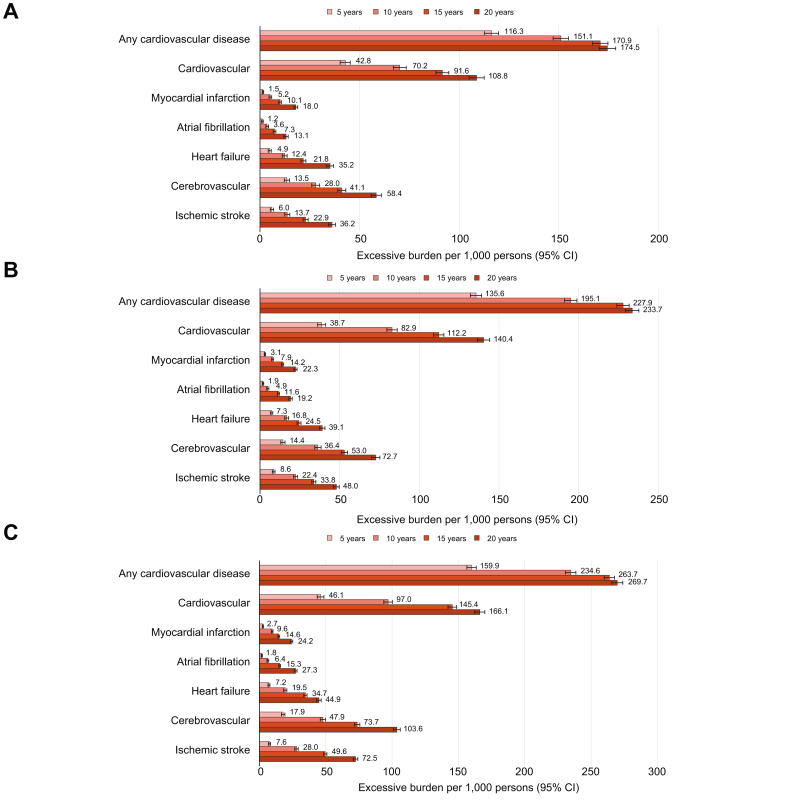
Excessive burdens of cardiovascular diseases by steatotic liver disease subtypes. (A) Metabolic dysfunction-associated steatotic liver disease. (B) Metabolic dysfunction-associated steatotic liver disease with increased alcohol consumption. (C) Alcohol-related liver disease.

We estimated the excessive burden of cardiovascular outcomes attributable to each SLD subtype – MASLD, MetALD, and ALD – at 5-, 10-, 15-, and 20-year follow-up intervals (Fig. 3A–C). The excessive burden was defined as the absolute difference in cumulative risk between each SLD group and the non-SLD reference group at each time point. In the MASLD group, the number of excess overall cardiovascular events per 1,000 individuals was 116.3 at 5 years, 151.1 at 10 years, 170.9 at 15 years, and 174.5 at 20 years, compared to non-SLD. These results indicate that the impact of MASLD on cardiovascular burden progressively increases with time. Among the outcomes, the excessive burden was consistently higher for cardiovascular diseases than for cerebrovascular diseases (Fig. 3A).

Similarly, the MetALD group demonstrated higher excessive burdens than the MASLD group (Fig. 3B). Among the three subtypes, the ALD group exhibited the greatest disease burden across all time points and all cardiovascular outcomes (Fig. 3C). The absolute risk differences in ALD remained substantially higher than those of the non-SLD group at 5-, 10-, 15-, and 20-year intervals. The trend was consistent across all event types, with cerebrovascular disease showing the most marked long-term increase in burden relative to non-SLD. These findings underscore the amplified impact of ALD on cardiovascular health over time.

### Relative risks of cardiovascular diseases for MASLD, MetALD, and ALD

Table 2 presents the adjusted RRs of cardiovascular diseases for each SLD subtype compared to the non-SLD group. MASLD was significantly associated with increased risk of any cardiovascular diseases, even after adjusting for age, sex, cigarette smoking, low-density lipoprotein-cholesterol, diabetes, blood pressure, FIB-4, and family history of cardiovascular diseases (*p <*0.05). The adjusted RR for any cardiovascular diseases in MASLD was 1.29 (1.28-1.31). For specific outcomes, the adjusted RRs ranged from 1.09 to 1.46 (all *p <*0.05).

**Table 2:** Relative risks of various cardiovascular diseases for MASLD, MetALD, and ALD compared to individuals without SLD.

Event	Subtype	Crude RR (95% CI)	Adjusted RR^a^ (95% CI)	Adjusted RR^b^ (95% CI)
Any cardiovascular diseases	MASLD	1.62 (1.61-1.64)	1.43 (1.41-1.45)	1.29 (1.28-1.31)
	MetALD	1.85 (1.80-1.90)	1.61 (1.57-1.66)	1.38 (1.34-1.42)
	ALD	2.04 (1.98-2.10)	1.75 (1.70-1.81)	1.48 (1.43-1.53)
Cardiovascular	MASLD	1.48 (1.46-1.50)	1.26 (1.24-1.28)	1.16 (1.15-1.18)
	MetALD	1.60 (1.54-1.67)	1.40 (1.35-1.46)	1.25 (1.20-1.30)
	ALD	1.75 (1.67-1.82)	1.53 (1.46-1.60)	1.35 (1.29-1.41)
Cerebrovascular	MASLD	1.58 (1.54-1.61)	1.20 (1.18-1.23)	1.09 (1.07-1.12)
	MetALD	1.69 (1.59-1.79)	1.37 (1.29-1.45)	1.16 (1.09-1.24)
	ALD	1.99 (1.86-2.12)	1.67 (1.56-1.78)	1.39 (1.30-1.49)
Myocardial infarction	MASLD	2.56 (2.41-2.72)	1.91 (1.80-2.04)	1.46 (1.36-1.56)
	MetALD	3.03 (2.62-3.49)	1.86 (1.61-2.15)	1.25 (1.07-1.46)
	ALD	3.25 (2.78-3.80)	1.98 (1.69-2.32)	1.21 (1.02-1.44)
Atrial fibrillation	MASLD	1.57 (1.50-1.65)	1.14 (1.09-1.20)	1.09 (1.04-1.15)
	MetALD	1.79 (1.57-2.03)	1.28 (1.13-1.45)	1.18 (1.03-1.35)
	ALD	2.14 (1.87-2.44)	1.60 (1.40-1.83)	1.42 (1.23-1.64)
Heart failure	MASLD	1.81 (1.75-1.88)	1.33 (1.29-1.38)	1.20 (1.16-1.25)
	MetALD	1.90 (1.73-2.07)	1.58 (1.44-1.73)	1.34 (1.21-1.47)
	ALD	2.10 (1.91-2.32)	1.86 (1.68-2.05)	1.47 (1.32-1.63)
Ischemic stroke	MASLD	1.81 (1.75-1.87)	1.34 (1.30-1.39)	1.15 (1.11-1.19)
	MetALD	2.10 (1.92-2.27)	1.60 (1.47-1.74)	1.24 (1.13-1.36)
	ALD	2.60 (2.38-2.84)	2.08 (2.48-2.54)	1.61 (1.47-1.77)

ALD, alcohol-associated liver disease; CMRF, cardiometabolic risk factor; MASLD, metabolic dysfunction-associated steatotic liver disease; MetALD, MASLD and increased alcohol consumption; RR, relative risk.

Levels of significance: *p* <0.05 for all results (Wald test).

a Adjusted for age and sex.

b Adjusted for age, sex, cigarette smoking, low-density lipoprotein-cholesterol, diabetes mellitus, blood pressure, Fibrosis-4 index, and family history of cardiovascular disease.

The magnitude of risk increased across the SLD spectrum. The adjusted RR was 1.38 (95% CI 1.34–1.42) for MetALD and 1.48 (95% CI 1.43–1.53) for ALD, respectively. Notably, the ALD group had the highest RR for most cardiovascular outcomes compared to non-SLD. However, for myocardial infarction, MASLD exhibited the strongest association, with an adjusted RR of 1.46 (95% CI 1.36–1.56), followed by MetALD at 1.25 (95% CI 1.07–1.46), and ALD at 1.21 (95% CI 1.02–1.44). These findings indicate that while all SLD subtypes elevate cardiovascular risk, MASLD remains a key contributor, particularly for myocardial infarction.

### Relative risks of cardiovascular diseases for MASLD, MetALD, and ALD after accounting for liver-related deaths as competing risks

To evaluate the robustness of our findings, we conducted competing risk analyses accounting for liver-related deaths, including cirrhosis and hepatocellular carcinoma. Table 3 presents both crude and adjusted SHRs for each SLD subtype. Including liver-related outcomes as competing risks did not alter the associations between SLD subtypes and cardiovascular diseases. For overall cardiovascular diseases, the adjusted SHRs remained elevated across all SLD subtypes when compared to non-SLD. MASLD was associated with a 1.62-fold increased risk of myocardial infarction (95% CI 1.51–1.73). Similar trends were observed for MetALD and ALD, with minor changes but persistent statistical significance for all major outcomes. These results reinforce the excessive cardiovascular risks associated with SLD subtypes.

**Table 3:** Subdistribution HRs of various cardiovascular diseases for MASLD, MetALD, and ALD, accounting for liver-related outcomes as competing risks.

Event	Subtype	Subdistribution HR (95% CI)	Subdistribution HR^a^ (95% CI)	Subdistribution HR^b^ (95% CI)
Any cardiovascular diseases	MASLD	1.63 (1.61-1.64)	1.43 (1.42-1.45)	1.29 (1.27-1.30)
	MetALD	1.81 (1.76-1.86)	1.57 (1.52-1.62)	1.34 (1.30-1.38)
	ALD	1.95 (1.89-2.01)	1.65 (1.59-1.71)	1.41 (1.36-1.46)
Cardiovascular	MASLD	1.48 (1.46-1.50)	1.26 (1.24-1.28)	1.18 (1.16-1.20)
	MetALD	1.59 (1.53-1.65)	1.37 (1.32-1.42)	1.24 (1.19-1.29)
	ALD	1.71 (1.64-1.78)	1.46 (1.40-1.53)	1.31 (1.25-1.37)
Cerebrovascular	MASLD	1.58 (1.55-1.62)	1.20 (1.17-1.23)	1.11 (1.08-1.13)
	MetALD	1.68 (1.58-1.78)	1.33 (1.25-1.41)	1.14 (1.07-1.22)
	ALD	1.93 (1.81-2.06)	1.56 (1.46-1.67)	1.31 (1.22-1.40)
Myocardial infarction	MASLD	2.58 (2.43-2.75)	1.94 (1.82-2.07)	1.62 (1.51-1.73)
	MetALD	3.04 (2.63-3.51)	1.86 (1.61-2.15)	1.27 (1.10-1.48)
	ALD	3.21 (2.74-3.77)	1.94 (1.65-2.28)	1.25 (1.06-1.47)
Atrial fibrillation	MASLD	1.57 (1.50-1.65)	1.13 (1.08-1.19)	1.08 (1.03-1.14)
	MetALD	1.77 (1.56-2.01)	1.24 (1.09-1.41)	1.12 (0.98-1.28)#
	ALD	2.08 (1.81-2.38)	1.49 (1.30-1.71)	1.32 (1.15-1.52)
Heart failure	MASLD	1.82 (1.75-1.88)	1.33 (1.29-1.38)	1.21 (1.17-1.26)
	MetALD	1.87 (1.70-2.04)	1.54 (1.40-1.69)	1.27 (1.15-1.40)
	ALD	2.05 (1.86-2.27)	1.78 (1.61-1.98)	1.41 (1.27-1.57)
Ischemic stroke	MASLD	1.82 (1.76-1.88)	1.33 (1.28-1.37)	1.17 (1.13-1.21)
	MetALD	2.07 (1.90-2.26)	1.53 (1.40-1.67)	1.21 (1.10-1.32)
	ALD	2.53 (2.32-2.77)	1.92 (1.75-1.10)	1.47 (1.34-1.62)

ALD, alcohol-associated liver disease; CMRF, cardiometabolic risk factor; HR, hazard ratio; MASLD, metabolic dysfunction-associated steatotic liver disease; MetALD, MASLD and increased alcohol consumption.

Levels of significance: #*p* = 0.0962, all other results *p* <0.05. (Wald test).

a Adjusted for age and sex.

b Adjusted for age, sex, cigarette smoking, low-density lipoprotein-cholesterol, diabetes mellitus, blood pressure, Fibrosis-4 index, and family history of cardiovascular disease.

### Population attributazble fractions of MASLD, MetALD, and ALD for cardiovascular diseases

Table 4 shows the PAFs of each SLD subtype associated with cardiovascular outcomes. After adjusting for covariates, MASLD was responsible for 8.07% (95% CI, 7.81-8.58) of all cardiovascular disease cases in the population. This includes 4.62% (95% CI, 4.34-5.17) of cardiovascular diseases and 2.65% (95% CI, 2.07-3.50) of cerebrovascular diseases. Among all outcomes, myocardial infarction had the highest PAF attributed to MASLD at 12.22% (95% CI 9.82–14.49).

**Table 4:** Population attributable fractions of steatotic liver disease subtypes for various cardiovascular diseases.

Event	MASLD			MetALD			ALD		
	PAF%∗	95% CI		PAF%∗	95% CI		PAF%∗	95% CI	
Any cardiovascular diseases	8.07	7.81	8.58	0.93	0.83	1.03	0.88	0.78	0.97
Cardiovascular	4.62	4.34	5.17	0.61	0.49	0.74	0.64	0.53	0.75
Cerebrovascular	2.65	2.07	3.50	0.39	0.22	0.59	0.71	0.55	0.89
Myocardial infarction	12.22	9.82	14.49	0.61	0.17	1.12	0.38	0.04	0.80
Atrial fibrillation	2.65	1.20	4.34	0.44	0.07	0.86	0.77	0.42	1.16
Heart failure	5.71	4.62	7.03	0.83	0.52	1.15	0.86	0.59	1.15
Ischemic stroke	4.34	3.22	5.44	0.59	0.32	0.88	1.11	0.86	1.40

ALD, alcohol-associated liver disease; CMRF, cardiometabolic risk factor; MASLD, metabolic dysfunction-associated steatotic liver disease; MetALD, MASLD and increased alcohol consumption; PAF, population attributable fraction.

∗ Adjusted for age, sex, cigarette smoking, low-density lipoprotein-cholesterol, diabetes mellitus, blood pressure, Fibrosis-4 index, and family history of cardiovascular disease.

Although the prevalence of MetALD and ALD was substantially lower, their associated risks still translated into measurable population burdens. The PAF for MetALD was 0.93% for any cardiovascular disease, with subtype-specific values ranging from 0.44% to 0.83% across major adverse cardiovascular event outcomes. ALD demonstrated a comparable PAF of 0.88% (95% CI 0.78–0.97) but exceeded MetALD in most major adverse cardiovascular event categories, except for myocardial infarction, where MASLD remained dominant. Collectively, these data illustrate that while MASLD contributes the largest share of the population burden, MetALD and ALD also represent non-negligible contributors to cardiovascular morbidity.

## Discussion

This large-scale prospective cohort study comprehensively evaluated the impact of MASLD on a range of cardiovascular outcomes. Our findings demonstrated a significant association between MASLD and elevated risks of these cardiovascular diseases. By quantifying the PAF, we showed a considerable proportion of cardiovascular events could potentially be prevented through effective management of MASLD, particularly by targeting modifiable cardiometabolic risk factors. Beyond MASLD, we extended our analysis to include the full spectrum of SLD, including MetALD and ALD, providing a more complete picture of how varying degrees of alcohol intake influence cardiovascular risk. These insights offer a valuable foundation for informing the clinical management and risk stratification of individuals across SLD subtypes.

Although the terminology has evolved from non-alcoholic fatty liver disease to MASLD, the diagnostic overlap remains substantial, with nearly 90% of individuals with intrahepatic triglyceride content above 5% meeting the criteria under both definitions.^28^ The updated nomenclature further delineates the SLD spectrum into distinct subtypes, including MASLD, MetALD, and ALD, based on alcohol consumption and cardiometabolic status. In our study, we applied the Asian-Pacific guideline^22^ to define limited alcohol intake (<20 g/day for men and <10 g/day for women), although clinical consensus guidelines recommend a higher cut-off (<30 g/day for men and <20 g/day for women) when classifying SLD subtypes.^23^ Notably, ALD accounted for approximately 5% of all SLD participants in our cohort. This relatively low prevalence may reflect genetic variants common in East Asian populations that affect alcohol metabolism, particularly variants in *ALDH2* and *ADH1B*, which result in unpleasant reactions to alcohol and are associated with reduced alcohol intake.^29^^,^^30^ Our findings demonstrated a stepwise increase in cardiovascular risk across the SLD subtypes, with the highest risk observed in ALD. Interestingly, however, the risk of myocardial infarction was greatest in the MASLD group, consistent with a recent Korean population-based study,^31^ suggesting that non-alcoholic metabolic dysfunction may have a particularly strong association with atherothrombotic events.

Previous meta-analyses have reported increased risks of composite fatal and non-fatal cardiovascular events in patients with MASLD,^13^ yet systematic data on specific subtypes like carotid atherosclerosis or stroke have been sparse. A recent study found that at least 35% of patients with MASLD had carotid atherosclerosis, and 5% had a history of stroke.^32^ Patients with more severe steatosis, as determined by liver pathology, also exhibited significant increases in mean carotid intima-media thickness.^32^ Our findings are consistent with a Chinese community-based prospective study that reported a 16% increased risk of ischemic stroke in patients with MASLD,^33^ a finding supported by a Caucasian study indicating a 1.26-fold increased risk.^34^ Notably, MASLD was associated with ischemic stroke but not hemorrhagic stroke.^32^^,^^34^

The variability in findings regarding the association between steatosis and cardiovascular subtypes may be attributed to differences in study populations, study designs, covariate adjustments, and diagnostic methods for steatosis. In our study, myocardial infarction emerged as the most frequent cardiovascular subtype, accounting for nearly 35% of cases. In our study, MASLD significantly increased myocardial infarction, aligning with findings from a Korean population-based study.^31^^,^^35^ This association was observed even in young adults aged 20-39 years,^36^ though a European study did not find a similar link.^37^ Regarding heart failure, our results align with a recent large-scale meta-analysis of 11 million middle-aged individuals.^38^ For atrial fibrillation, a previous study has suggested a connection with steatosis.^17^ Over a 10-year follow-up, patients with fatty liver disease experienced an absolute risk increase of 1.3 per 1,000 person-years for atrial fibrillation compared to patients without fatty liver disease,^17^ which is comparable to our finding of 2.4 per 1,000 person-years. The association between MASLD and atrial fibrillation was weaker, with an RR of 1.09. Interestingly, the Rotterdam study found no association,^16^ likely due to the older age (mean 65 years) and higher comorbidity burden of its population. Variations across studies arise from differences in methodology: some combine fatal and non-fatal events,^37^ while others diagnose fatty liver using seromarkers^31^^,^[34], [35], [36] or transient elastography.^16^ Additionally, variations in lifestyle habits across ethnic groups may contribute to the differing associations between steatosis and cardiovascular diseases.

Recent evidence suggests that MASLD contributes to cardiovascular disease through multiple interrelated mechanisms, including lipotoxicity, insulin resistance, oxidative stress, and systemic inflammation.[39], [40], [41] Hepatic inflammation in MASLD triggers the release of a wide range of proinflammatory mediators, leading to endothelial dysfunction, vascular injury, and atherogenesis.^41^ Activation of pathways, such as the NF-κB signaling, plays a central role in amplifying inflammatory responses and promoting both hepatic and systemic insulin resistance.^39^^,^^42^ Approximately 20% of MASLD cases progress to metabolic dysfunction-associated steatohepatitis, which is characterized by elevated systemic inflammatory markers such as high-sensitivity C-reactive protein and CXCL10.[43], [44], [45] Additionally, steatotic hepatocyte-derived extracellular vesicles have been shown to promote foam cell formation and accelerate atherosclerosis, further linking liver-derived inflammation with cardiovascular pathology.^40^

Given the associations between steatotic liver diseases (MASLD, MetALD, and ALD) and cardiovascular diseases, improving cardiovascular risk assessment in individuals with SLD is essential. Although many cardiovascular risk prediction models exist,^46^^,^^47^ few have been specifically validated in SLD populations. Recent large-scale studies suggest that commonly used models tend to overestimate the risk of myocardial infarction and coronary artery diseases, especially in low-risk individuals.^48^ Considering the elevated cardiometabolic burden in SLD, it is important to evaluate and tailor these prediction models for use in this population. Additionally, current risk calculators do not include SLD as an independent cardiovascular disease risk factor. However, increasing disease severity, particularly when steatohepatitis or liver fibrosis is present, has been linked to higher cardiovascular risk.^49^ Incorporating liver-specific indicators into cardiovascular risk models may improve their accuracy and help guide more effective prevention strategies.

Owing to the limited epidemiological data on MASLD and its long-term association with cardiovascular events, we performed this large, well-characterized cohort study, which enabled detailed analyses accounting for a wide range of sociodemographic, clinical, and cardiometabolic risk factors. The extended follow-up period enabled sufficient time for cardiovascular disease development, with reliable event ascertainment through nationwide registries. As participants were generally healthy individuals undergoing health examinations, these findings are relevant to cardiovascular prevention strategies in comparable populations. MASLD was diagnosed using abdominal ultrasonography, a clinically applicable method for detecting hepatic steatosis. While biochemical indices such as the fatty liver index are also validated and widely used in population-based studies with reported positive predictive values of up to 99%,^50^ ultrasonography remains a widely applicable and feasible modality for both clinical practice and population-based screening. However, we acknowledge that ultrasonography may misclassify mild steatosis, potentially resulting in misclassification of some individuals as non-SLD. This underestimation could bias our findings toward the null, suggesting that the true associations between SLD and cardiovascular events may be even stronger than reported.

In conclusion, MASLD significantly increases the risk of major cardiovascular events, accounting for an estimated 8.1% of the disease burden. While MASLD remains the primary contributor, both MetALD and ALD are associated with elevated risks, with ALD showing the highest risk. These findings emphasize the need to integrate SLD subtypes, especially MASLD, into cardiometabolic prevention strategies to reduce long-term cardiovascular risks.

## Abbreviations

ALD, alcohol-associated liver disease; MASLD, metabolic dysfunction-associated steatotic liver disease; MetALD, MASLD and increased alcohol intake; PAF, population attributable fraction; SLD, steatotic liver disease; RR, relative risk.

## Financial support

This study was supported by the National Science and Technology Council, Taipei, Taiwan (grant: 112-2628-B-A49-007 and 113-2628-B-A49-012), the Higher Education Sprout Project by the Ministry of Education (MOE) in Taiwan, and by the National Health Research Institute, Chunan, Taiwan (grant: NHRI-EX112-11117PI). None of the funding organizations contributed to the study design and delivery, data collection, management, analysis, and interpretation, data preparation and review, or manuscript approval.

## Authors’ contributions

Study concept and design: Mei-Hsuan Lee; acquisition of data: Mei-Hsuan Lee; analysis and interpretation of data: Szu-Ching Yin and Mei-Hsuan Lee; drafting of the manuscript: Szu-Ching Yin and Mei-Hsuan Lee; critical revision of the manuscript for important intellectual content: Szu-Ching Yin, Yi-Ting Chen, Wei-Ting Chang, Tzu-I Chen, Tsai-Hsuan Yang, Xia-Rong Liu, Chia-Wei Huang, Yu-Wei Chen, Mei-Hsuan Lee; acquisition of funding and study supervision: Mei-Hsuan Lee.

## Data availability statement

All or part of the data used in this research were authorized by and received from MJ Health Research Foundation (Authorization Code: MJHRF2022001A) and Health and Welfare Data Science Center Database, Ministry of Health and Welfare (NHIRD_MOHW: H111164). Dr. Mei-Hsuan Lee applied for all data use. Other researchers may request the materials used through collaboration.

## Conflict of interest

The authors of this study declare that they do not have any conflict of interest.

Please refer to the accompanying ICMJE disclosure forms for further details.
