# Supplementary material for: Attributable burden of steatotic liver disease on cardiovascular outcomes in Asia
Source: JHEP Rep. 2025 Jun 6;7(9):101479. doi: 10.1016/j.jhepr.2025.101479 (PMC12341586; doi:10.1016/j.jhepr.2025.101479)
Supplement: Multimedia component 1 [file mmc1.pdf]

Attributable burden of steatotic liver disease on cardiovascular outcomes in Asia

Szu-Ching Yin, Yi-Ting Chen, Wei-Ting Chang, Tzu-I Chen, Tsai-Hsuan Yang, Xia-Rong Liu, Chia-Wei Huang, Yu-Wei Chen, Mei-Hsuan Lee

Table of contents

Table S1……………………………………………………………………………2

Table S2……………………………………………………………………………3

Fig. S1………………………………………………………………………………5

Table S1. International Classification of Diseases (ICD) codes for cardiovascular diseases.

Events	ICD-9	ICD-10
**Any cardiovascular diseases**	390-459	I00-I99
**Cardiovascular disease**	410-414; 420-429	I20-I25; I30-I52
**Myocardial infarction**	410	I21-I23
**Atrial fibrillation**	427.31, 427.32	I48
**Heart failure**	428	I50
**Cerebrovascular disease**	430-438	I60-I69
**Ischemic stroke**	433, 434	I63

Table S2. Incidence rates on various cardiovascular diseases according to steatotic liver disease subtypes.

Events	SLD subtypes	Number of events	Person-years of follow-up	Incidence rate per 100,000 person-years
**Any cardiovascular diseases**	Non-SLD	96,302	2326077.2	4140.1
	MASLD	57,455	831503.6	6909.8
	MetALD	5,154	65256.0	7898.1
	ALD	4,048	46296.4	8743.7
**Cardiovascular**	Non-SLD	51,773	2934371.0	1764.4
	MASLD	31,332	1198221.3	2614.9
	MetALD	2,787	98484.2	2829.9
	ALD	2,224	72,166	3081.8
**Cerebrovascular**	Non-SLD	19,159	3320581.5	577.0
	MASLD	12,745	1424715.6	894.6
	MetALD	1,134	117590.4	964.4
	ALD	983	86760.9	1133.0
**Myocardial infarction**	Non-SLD	1,935	3478266.5	55.6
	MASLD	2,096	1528179.7	137.2
	MetALD	208	126176.7	164.8
	ALD	168	94152.0	178.4
**Atrial fibrillation**	Non-SLD	4,038	3465320.1	116.5
	MASLD	2,674	1524381.7	175.4
	MetALD	256	126058.3	203.1
	ALD	230	93809.3	245.2

**Heart failure**	Non-SLD	7,570	3436102.5	220.3
	MASLD	5,812	1497706.5	388.1
	MetALD	510	124084.9	411.0
	ALD	422	92170.0	457.8
**Ischemic stroke**	Non-SLD	7,848	3427949.7	228.9
	MASLD	6,045	1492168.4	405.1
	MetALD	580	122954.1	471.7
	ALD	534	91047.6	586.5

Non-SLD, non-steatotic liver disease; MASLD, metabolic dysfunction-associated steatotic liver disease; MetALD, MASLD and increased alcohol consumption; ALD, alcohol-associated liver disease; CMRF, cardiometabolic risk factor; RR, relative risk; CI, confidence interval.

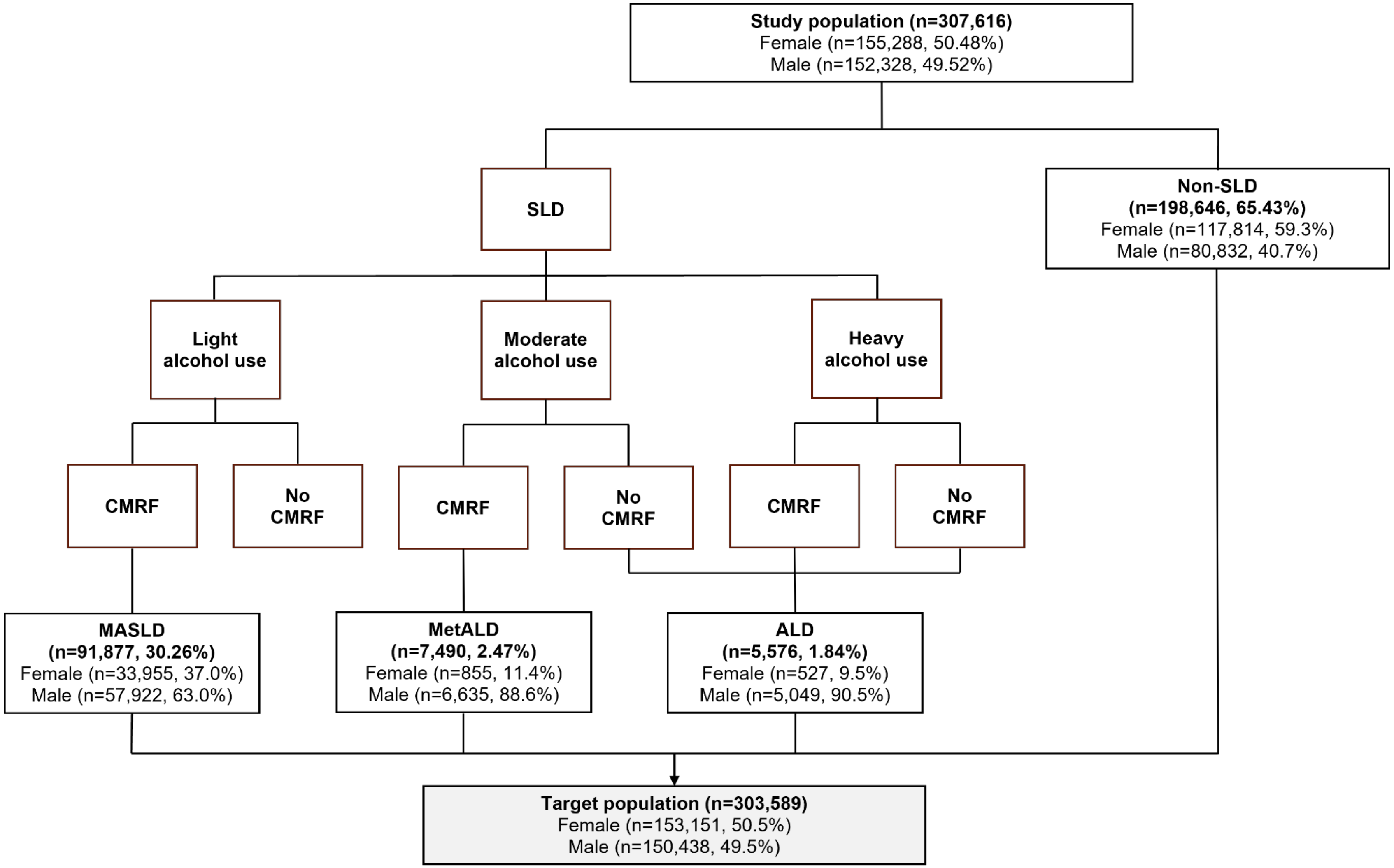

Study population (n=307,616)
Female (n=155,288, 50.48%)
Male (n=152,328, 49.52%)
SLD
Non-SLD
(n=198,646, 65.43%)
Female (n=117,814, 59.3%)
Male (n=80,832, 40.7%)
Light alcohol use
Moderate alcohol use
Heavy alcohol use
CMRF
No CMRF
CMRF
No CMRF
CMRF
No CMRF
MASLD
(n=91,877, 30.26%)
Female (n=33,955, 37.0%)
Male (n=57,922, 63.0%)
MetALD
(n=7,490, 2.47%)
Female (n=855, 11.4%)
Male (n=6,635, 88.6%)
ALD
(n=5,576, 1.84%)
Female (n=527, 9.5%)
Male (n=5,049, 90.5%)
Target population (n=303,589)
Female (n=153,151, 50.5%)
Male (n=150,438, 49.5%)

Fig. S1.
