# Supplementary material for: Attributable burden of steatotic liver disease on cardiovascular outcomes in Asia
Source: JHEP Rep. 2025 Jun 6;7(9):101479. doi: 10.1016/j.jhepr.2025.101479 (PMC12341586; doi:10.1016/j.jhepr.2025.101479)
Supplement: Multimedia component 2 [file mmc2.docx]

**JHEP Reports**

**CTAT methods**

Tables for a “Complete, Transparent, Accurate and Timely account” (CTAT) are now mandatory for all revised submissions. The aim is to enhance the reproducibility of methods.

- Only include the parts relevant to your study
- Refer to the CTAT in the main text as ‘Supplementary CTAT Table’
- Do not add subheadings
- Add as many rows as needed to include all information
- Only include one item per row

**If the CTAT form is not relevant to your study, please outline the reasons why:**

| This study involved secondary analysis of anonymized health records from a large-scale electronic health database. No laboratory experiments, biological materials, antibodies, cell lines, or reagents were used. The CTAT table is therefore not applicable. |
| --- |

- 1. **Antibodies**

| **Name** | **Citation** | **Supplier** | **Cat no.** | **Clone no.** |
| --- | --- | --- | --- | --- |
|  |  |  |  |  |

- 1. **Cell lines**

| **Name** | **Citation** | **Supplier** | **Cat no.** | **Passage no.** | **Authentication test method** |
| --- | --- | --- | --- | --- | --- |
|  |  |  |  |  |  |

- 1. **Organisms**

| **Name** | **Citation** | **Supplier** | **Strain** | **Sex** | **Age** | **Overall n number** |
| --- | --- | --- | --- | --- | --- | --- |
|  |  |  |  |  |  |  |

- 1. **Sequence based reagents**

| **Name** | **Sequence** | **Supplier** |
| --- | --- | --- |
|  |  |  |

- 1. **Biological samples**

| **Description** | **Source** | **Identifier** |
| --- | --- | --- |
|  |  |  |

- 1. **Deposited data**

| **Name of repository** | **Identifier** | **Link** |
| --- | --- | --- |
| **MJ Health Research Database** | **Not publicly available** | **http://www.mjhrf.org/en/index.php** |

- 1. **Software**

| **Software name** | **Manufacturer** | **Version** |
| --- | --- | --- |
| **SAS** | **SAS Institute Inc., Cary, NC** | **9.4** |

- 1. **Other (*e.g*. drugs, proteins, vectors etc.)**

|  |  |  |
| --- | --- | --- |
|  |  |  |

- 1. **Please provide the details of the corresponding methods author for the manuscript:**

| **Mei-Hsuan Lee**  **Institute of Clinical Medicine, National Yang Ming Chiao Tung University**  **155 Li-Nong Street, Section 2, Beitou, Taipei 112, Taiwan**  **Tel: +886-2-2826-7248**  **Fax: +886-2-2820-5699**  **E-mail: meihlee@nycu.edu.tw** |
| --- |

**2.0 Please confirm for randomised controlled trials all versions of the clinical protocol are included in the submission. These will be published online as supplementary information.**

|  |
| --- |
